# Analysis of Korean Retinal Specialists’ Opinions on Implanting Diffractive Multifocal Intraocular Lenses in Eyes with Underlying Retinal Diseases

**DOI:** 10.3390/jcm11071836

**Published:** 2022-03-26

**Authors:** Jung-Hwa Lee, Mingui Kong, Joon-Hong Sohn, Beom-Jin Cho, Kee-Yong Choi, Sang-Mok Lee

**Affiliations:** 1 Department of Ophthalmology, HanGil Eye Hospital, 35 Bupyeong-daero, Bupyeong-gu, Incheon 21388, Korea; bibacoomon@naver.com (J.-H.L.); eyedockong@gmail.com (M.K.); jhsohn19@nate.com (J.-H.S.); chobjn@empal.com (B.-J.C.); cky104@empal.com (K.-Y.C.); 2 Department of Ophthalmology, Catholic Kwandong University College of Medicine, 24 Beomil-ro 579 beon-gil, Gangneung-si 25601, Korea

**Keywords:** cataract, multifocal intraocular lenses, retinal diseases, macular degeneration, diabetic retinopathy, guidelines, surveys and questionnaires

## Abstract

Multifocal intraocular lenses (MF-IOLs) are increasingly implanted as the need for good near- and intermediate-distance vision increases. Although retinal disease is known to be a relative contraindication for MF-IOL implantation, there are no detailed guidelines for MF-IOL implantation with respect to the type and severity of retinal diseases/statuses. In this study, because retinal diseases can affect the performance of MF-IOLs, we analyzed the opinions of 111 retinal specialists, who were members of the Korean Retina Society, on the implantation of diffractive MF-IOLs in eyes with 15 retinal diseases/statuses using a web-based survey. For each underlying condition, retinal specialists were asked to rate their approval regarding implantation of MF-IOLs on a scale from 1 (completely disapprove) to 7 (completely approve), under the assumption that there were no known contraindications except for a given retinal disease/status. As a result, retinal specialists disapproved MF-IOL implantation (median value of Likert score < 4) in the eyes with wet age-related macular degeneration, dry age-related macular degeneration with geographic atrophy, proliferative diabetic retinopathy, nonproliferative diabetic retinopathy with macular edema, previous macula-off retinal detachment, previous retinal vein occlusion, and epiretinal membrane, but the scores varied by disease/status. The factors that affected the specialists’ opinions were the type of practice and the frequency of MF-IOL implantation (*p* = 0.013 and *p* = 0.021, respectively; one-way ANOVA).

## 1. Introduction

As the use of digital devices has increased, good near- and intermediate-distance vision has become an increasingly important issue even for elderly individuals [1]. However, conventional monofocal intraocular lens (IOL) implantation during cataract surgery cannot restore the accommodation capacity and may further reduce capacity, especially in younger patients. Multifocal intraocular lens (MF-IOL) implantation has been recognized as the most efficient and safe method of improving spectacle independence and near-vision expectations in comparison to other available methods, and its use is increasing [2,3]. According to the results of an annual survey of Korean cataract surgeons, the percentage of surgeons who consider MF-IOL implantation for cataract surgery if indicated has increased from 44% in 2012 to 76% in 2018 [4]. Although there are no official data, according to IOL import data and government monitoring data [5], the market share of MF-IOL in Korea in 2020 is expected to be around 35%.

MF-IOL makes two or more focal points, thereby improving near and/or intermediate vision. Of the diverse designs of MF-IOLs, diffractive IOLs generate multifocality based on light interference and the spreading of waves around obstacles [6,7]. A series of annular concentric grooves carved around the optical axis can create a wave spread in numerous directions, which can reinforce or cancel each other as they overlap, creating multiple focal points according to the unique optical design [6,7]. However, diffractive MF-IOLs are known to be associated with lower contrast sensitivity in comparison to monofocal IOLs, especially in mesopic conditions, which may be due to the superposition of one sharp image with one or more out-of-focus images [7,8]. An increased incidence of dysphotopsia has been reported in MF-IOL-implanted eyes, which correlates with more intraocular scattered light [8].

Comorbid retinal disease has been reported in approximately 22% to 31% of patients who have undergone cataract surgery [9,10]. Patients with macular disorders, such as age-related macular degeneration (AMD) or central serous chorioretinopathy (CSC), often complain of unsatisfactory vision quality due to reduced contrast sensitivity despite objectively good vision [2]. Macular disease is known as a relative contraindication for MF-IOL implantation, considering the possible synergistic effect of both conditions on reducing contrast sensitivity especially under low-illumination conditions and the increased long-term risk of developing late-stage, age-related maculopathy after cataract surgery [11,12,13,14]. However, the clinical outcomes of MF-IOL implantation in eyes with retinal diseases, including macular disorders, have not been sufficiently studied, and there are no evidence-based guidelines for the implantation of MF-IOLs regarding the type and severity of retinal disease.

Because of the challenges of prospective clinical trials for this population, this study aims to provide information to help cataract surgeons make decisions about implanting diffractive MF-IOLs in eyes with underlying retinal diseases by summarizing the opinions of retinal specialists.

## 2. Methods

### 2.1. Participants

The study was conducted with the cooperation of the Korean Retina Society (KRS) between March 2019 and April 2019 using email-distributed surveys linked to SurveyMonkey.com (San-Mateo, CA, USA). Members of the KRS were invited by e-mail to participate in the survey. The KRS consists of qualified experts who have completed a retinal fellowship that lasted more than a year and published two or more papers on the retina as a first or corresponding author during the training period. Of the 364 KRS members in 2019, 111 specialists completed the survey (30.5%). Participation in the study was voluntary, responses were confidential, data were submitted anonymously, and no incentives were provided for participation. The participants were informed of the purpose of the study in the cover letters of the questionnaires. The study adhered to the tenets of the Declaration of Helsinki and was considered exempt from the institutional review board and ethical committee at the HanGil Eye Hospital since only expert opinions regarding the implantation of MF-IOLs in eyes with retinal diseases were collected, and no patient data were used.

### 2.2. Selection of the Underlying Retinal Diseases/Statuses for the Survey

The survey aimed to obtain the opinions of Korean retinal specialists on implanting MF-IOLs in eyes with diverse underlying retinal diseases/statuses considering the potential effect of implanting MF-IOLs on the diagnosis and treatment of underlying diseases. Based on previous literature and the results of a preliminary survey, fifteen retinal diseases/statuses were chosen by the authors (M.K. and J.-H.S.). The preliminary survey included open-ended essay questions about the challenges of diagnosing and treating retinal diseases in eyes with MF-IOLs. The preliminary survey was distributed to retinal specialists by e-mail between August and October 2018 and received 32 responses. Based on these responses, 15 retinal diseases/statuses were chosen for the survey, namely (1) nonproliferative diabetic retinopathy (NPDR) without macular edema (ME); (2) NPDR with ME; (3) proliferative diabetic retinopathy (PDR) without ME; (4) PDR with ME; (5) resolved CSC; (6) previous retinal vein occlusion (RVO); (7) dry AMD with geographic atrophy (GA); (8) dry AMD without GA; (9) wet AMD; (10) epiretinal membrane; (11) high myopia; (12) previous macula-on retinal detachment (RD); (13) previous macula-off RD; (14) lattice degeneration; and (15) a history of barrier laser photocoagulation due to retinal breaks.

### 2.3. Survey

**Figure 1:**
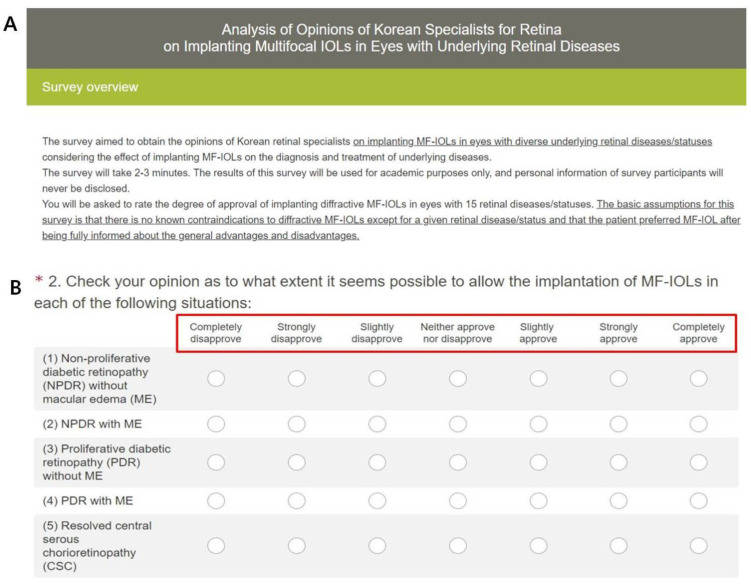
(**A**) The overview for the survey on SurveyMonkey. The introductory summary presented at the top of the survey is designed to introduce the time required to complete the survey and the basic assumptions of the survey that may help respondents answer the questions. (**B**) Examples of the survey format on SurveyMonkey. This screen capture shows the actual survey form for the first 5 of a total of 15 retinal diseases/statuses. The opinions of Korean retinal specialists on implanting diffractive multifocal intraocular lenses in eyes with specified retinal diseases were collected. Participants were asked to answer each question on a 7-point Likert scale, where a rating of 1 means “completely disapprove”; 2, “strongly disapprove”; 3, “slightly disapprove”; 4, “neither approve nor disapprove” (neutral); 5, “slightly approve”; 6, “strongly approve”; and 7, “completely approve”.

The survey was created, completed, and submitted on SurveyMonkey.com. It was designed to be completed within 5 min to encourage participation. The survey was performed under the basic assumption that there was no known contraindication to diffractive MF-IOLs except for a given retinal disease/status and that the patient preferred MF-IOL after being fully informed about the general advantages and disadvantages. In the survey, the experts were asked to use a 7-point Likert scale to rate their degree of approval of diffractive MF-IOLs in eyes with each of the 15 retinal diseases/statuses that might affect the performance of MF-IOLs; a rating of 1 meant “completely disapprove”; 2, “strongly disapprove”; 3, “slightly disapprove”; 4, “neither approve nor disapprove” (neutral); 5, “slightly approve”; 6, “strongly approve”; and 7, “completely approve” (Figure 1).

At the end of the survey, the specialists’ age group, career duration, type of practice, and frequency with which they implanted MF-IOLs were collected to analyze the factors that might affect specialists’ opinions.

### 2.4. Analysis of Opinions

The median values of the specialists’ opinions on each retinal disease/status were analyzed. Then, the percentage of permissive opinions was analyzed, with ratings from 5 (slightly approve) to 7 (completely approve) defined as permissive. In contrast, ratings from 1 (completely disapprove) to 3 (slightly disapprove) were defined as indicating disapproval of the procedure.

Analysis of the factors affecting the opinions was performed by adding all the scores for the 15 retinal diseases/statuses per respondent and categorizing the scores based on specialist age (30 s, 40 s, or 50–60 s), career duration (<10 years or ≥10 years), type of practice (tertiary hospital, eye hospital, or private eye clinic), and frequency of MF-IOL implantation (none, ≤5% of cases, or >5% of cases).

### 2.5. Statistical Analysis

The Mann–Whitney U test and one-way ANOVA were used to analyze the tendency of opinions according to the characteristics of the respondents, namely their age, career duration, type of practice, and frequency of MF-IOL implantation. All statistical analyses were performed using IBM SPSS Statistics (version 21; IBM Corp, New York, NY, USA), and *p* < 0.05 was considered to indicate statistical significance.

## 3. Results

Of the KRS members, 30.5% participated in this survey. The characteristics of the survey respondents are shown in Table 1.

**Figure 2:**
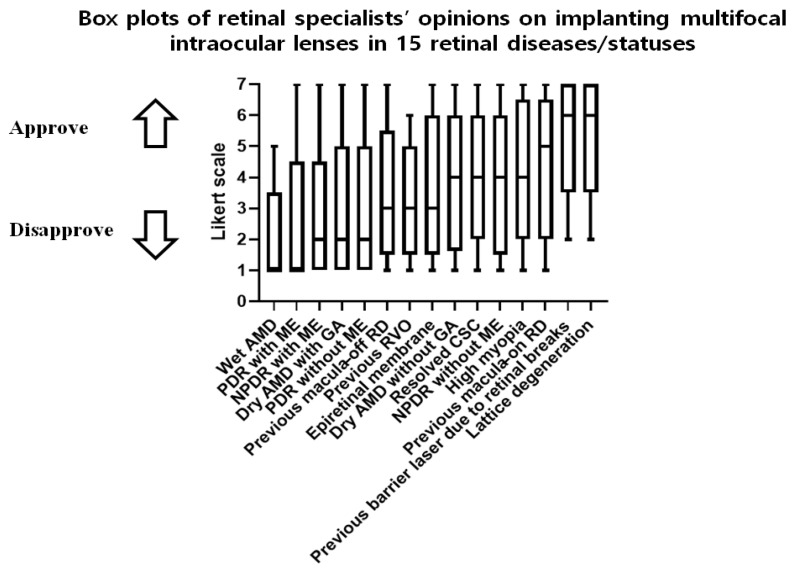
Box plots of the opinions of Korean retinal specialists on implanting diffractive MF-IOLs in eyes with 15 retinal diseases/statuses. Korean retinal specialists’ opinions on implanting MF-IOLs, reported on a 7-point Likert scale; the minimum, 1st quartile (25%, lower limit of box), median value (50%, middle line in box), 3rd quartile (75%, upper limit of box), and maximum are presented in a box plot for each retinal disease/status. The respondents used a 7-point Likert scale, where a rating of 1 means “completely disapprove”; 2, “strongly disapprove”; 3, “slightly disapprove”; 4, “neither approve nor disapprove”; 5, “slightly approve”; 6, “strongly approve”; and 7, “completely approve”. AMD, age-related macular degeneration; CSC, central serous chorioretinopathy; GA, geographic atrophy; NPDR, nonproliferative diabetic retinopathy; ME, macular edema; MF-IOL, multifocal intraocular lens; PDR, proliferative diabetic retinopathy; RD, retinal detachment; RVO, retinal vein occlusion.

The Korean retinal specialists’ recommendations regarding MF-IOLs based on a given retinal disease/status are summarized in Table 2 and Figure 2. The specialists’ opinions, arranged by median value from 1 (completely disapprove) to 7 (completely approve), were as follows: the specialists completely disapproved of implanting MF-IOLs in eyes with wet AMD and PDR with ME; strongly disapproved of implantation in eyes with NPDR with ME, dry AMD with GA, and PDR without ME; and slightly disapproved of implantation in eyes with previous macula-off RD, previous RVO, and epiretinal membrane. In contrast, the specialists were neutral regarding implantation in eyes with dry AMD without GA, resolved CSC, NPDR without ME, and high myopia; slightly approved of implantation in eyes with previous macula-on RD; and strongly approved of implantation in eyes with a history of barrier laser due to retinal breaks and lattice degeneration (Table 2).

Regarding the distribution of opinions, less than 30% were within the permissive range for the implantation of MF-IOLs in eyes with wet AMD, PDR with/without ME, NPDR with ME, dry AMD with GA, and previous history of macula-off RD/RVO. On the other hand, most of the specialists were not reluctant to implant MF-IOLs in eyes with a history of barrier laser due to retinal breaks (82.0%) or in eyes with lattice degeneration (82.9%).

The average sums of the questionnaire scores in subgroups defined by age, career duration, type of practice, and frequency of MF-IOL implantation are analyzed in Table 3. Among the factors that might affect specialists’ opinions, the type of practice and frequency of MF-IOL implantation showed a significant influence on opinions (*p* = 0.014 and *p* = 0.021, respectively; one-way ANOVA). However, age group and career duration did not significantly affect the opinions.

## 4. Discussion

In this study, retinal specialists expressed disapproval of MF-IOL implantation in eyes with wet AMD, PDR regardless of ME, NPDR with ME, dry AMD with GA, previous macula-off RD, previous RVO, and epiretinal membrane. Relatively permissive opinions were expressed regarding MF-IOL implantation in eyes with retinal diseases/statuses that did not prominently involve the macula, including previous macula-on RD, previous history of barrier laser, and lattice degeneration. Neutral opinions were expressed for implantation in eyes with dry AMD without GA, resolved CSC with relatively intact outer-retinal structures, NPDR without ME, and high myopia.

Because patients with macular disease often complain of unsatisfactory vision related to decreased contrast sensitivity despite objectively good visual acuity [2], the implantation of an MF-IOL, which can also reduce contrast sensitivity, into an eye with macular disease is considered a relative contraindication considering the possible synergistic effect of the two conditions [11,12,13,14]. However, given that the risk factors for retinal diseases and cataracts overlap considerably, it is unsurprising that these two diseases occur together frequently in the elderly population [15]. In a large study based at a public general hospital, approximately 20% of patients who had undergone cataract surgery were reported to have comorbid retinal disease, including age-related maculopathy (12.6%), diabetic retinopathy (9.0%), epiretinal membrane (2.9%), RD (0.8%), retinal artery/vein occlusion (0.8%), and macular holes (0.8%) [9]. In a prospective observational study of subjects undergoing cataract surgery, fundus examination revealed minimal age-related macular changes in 31% and significant retinal disease in 7.6% of eyes, including significant AMD, diabetic retinopathy with ME, macular holes, and previous RVOs [10]. Therefore, prior to cataract surgery, especially if MF-IOL implantation is planned, a more thorough preoperative retinal examination is required to determine whether comorbid retinal diseases are present; spectral-domain optical coherence tomography (OCT) is reported to be effective in detecting comorbid macular diseases that can be missed in a cloudy fundus view [3]. Because retinal diseases involving the macula can decrease contrast sensitivity, macular disease is regarded as a relative contraindication for the implantation of MF-IOLs in the same eye [2,14,16]. However, a recent paper presented doubts about this concept and argued that more research is needed based on (i) previous studies showing controversial results regarding the effect of MF-IOL on contrast sensitivity, (ii) contrast sensitivity levels that remained within the age-matched normal range with the MF-IOL even in the studies that showed significantly decreased contrast sensitivity with MF-IOLs compared to monofocal IOLs, and (iii) two reports that showed subjective patient satisfaction with MF-IOL implantation in eyes with concurrent retinal disease [7]. In this study, retinal specialists expressed disapproval of MF-IOL implantation in eyes with retinal diseases involving the macula, such as wet AMD, dry AMD with GA, PDR, NPDR with ME, previous macula-off RD, previous RVO, and epiretinal membrane; the percentage of disapproving opinions in these conditions was more than 50% (Table 2). However, among expert opinions on macula-related conditions, opinions on dry AMD without GA and resolved CSC were generally neutral, and approximately 35% to 41% of opinions on these conditions were permissive. Of the retinal diseases not involving the macula, only PDR without ME was a strongly disapproved condition for MF-IOL implantation; only 10.8% of opinions regarding this condition were permissive. This result is thought to have been influenced by the high likelihood of future ME in eyes with PDR.

The problems with MF-IOL implantation in eyes with retinal disease include not only the reduction in contrast sensitivity but also the effect on the diagnosis and treatment of preexisting retinal diseases [14,17,18,19,20,21]. Difficulties during retinal surgery in eyes with diffractive MF-IOL have been reported and include decreased contrast sensitivity for removal of the epiretinal membrane and skipped/ghost images of intravitreal triamcinolone crystals at some depth, which interfere with the intraoperative view [17,18]. In our preliminary survey, some retinal surgeons reported altered depth of focus during peeling of the internal limiting membrane, and the use of a wide-angle viewing system could alleviate this difficulty by altering optical paths, as reported in a recent article [22]. In this study, the median opinion regarding MF-IOL implantation in eyes with epiretinal membranes was “slightly disapprove”; 52.3% of the opinions were disapproving, possibly due to the idea that this difficulty can be overcome with an adequate viewing system and an increase in surgical experience with these situations. Recently, in the presence of MF-IOL in epiretinal surgery, it was reported that the surgical time was longer for the procedure to create a membrane edge or flap using retinal microforceps, but the surgical outcome was similar [23].

Confusion in interpreting examination results has also been reported in eyes with MF-IOLs. Wavy, horizontal artifacts have been reported on OCT line-scanning ophthalmoscopy images (monitor images) in eyes with diffractive MF-IOLs; however, there were no artifacts in OCT images or scanning laser ophthalmoscopy images [18]. Retinal OCT image quality has been reported to be significantly decreased by more than 3 dB in the MF-IOL group; however, there were no significant differences in measurements of macular thickness or volume [20,24]. There were also some comments on the decreased quality of the OCT images in our preliminary survey; however, most of the respondents reported that it was not critical for proper diagnosis. A significant nonspecific reduction in mean deviation values in Humphrey 10-2 visual-field testing was reported in eyes with MF-IOL, and this reduction did not recover until 6 months postoperatively [25].

Among the diverse factors that might affect specialists’ opinions, the type of practice and the frequency of MF-IOL implantation were associated with significant differences. In order to interpret these analysis results, it is necessary to understand the role of the retinal specialist (surgeon) in Korea. In Korea, previously, retinal specialists did not perform cataract surgery (about age over 60), but recently, as vitrectomy has become dominant, most retinal specialists perform cataract surgery combined with vitrectomy by themselves. Although some retinal surgeons often perform cataract surgery without vitrectomy, implantation of MF-IOL is not generally considered when considering the underlying disease of the eyes that retinal specialists mainly deal with. That is why the MF-IOL implantation frequency option was set relatively low (none, ≤5% of cases, or >5% of cases) in the questionnaire compared with usual proportion of MF-IOL implantation in Korea. In a previous survey of ophthalmologists regarding which IOLs they would choose for themselves, ophthalmologists who had performed more than 50 MF-IOL implantations were twice as likely to choose the MF-IOL option [26]. Although we did not ask about the absolute amount of experience with MF-IOL implantation, similar results were observed because the high frequency of MF-IOL implantation was associated with significantly permissive opinions on MF-IOL implantation in eyes with retinal diseases (*p* = 0.021) (Table 3). Furthermore, in Korea, retinal specialists at tertiary hospitals focus more on retinal surgery itself, whereas retinal specialists at private clinics focus on cataract surgery rather than retinal surgery even though they mainly deal with eyes with retinal diseases. It was reported that in Korea, premium IOL, including MF-IOL and toric monofocal IOL, was used with the highest frequency of 40.8% at the private clinic level in 2020 [5]. In these aspects, the type of practice, which can affect a specialist’s level of experience with MF-IOLs, was also identified as a significant factor related to the opinion of the retinal specialists (*p* = 0.013), with more disapproving opinions associated with working in a tertiary hospital. Age and career duration were added to the survey items because they were expected to be related in terms of whether cataract surgery was performed by themselves, as discussed above, and possible conservative view on the newly developed IOL in older age group, but they did not have a significant effect on opinions on implanting MF-IOL in eyes with retinal diseases.

There are several limitations to our study. First, our survey focused only on diffractive MF-IOLs and excluded newly developed MF-IOLs with sectoral refractive design and extended depth of focus design because it takes time for retinal specialists to acquire sufficient experience with new types of IOLs. Opinions on diffractive MF-IOLs have already been based on more than 10 years of experience, while other newly designed types, which are expected to be more tolerable for retinal diseases, have relatively short experience. Second, the survey response rate was only 30.5%. However, due to the limitations of the web-based, e-mail-distributed survey method, it was difficult to increase the response rate beyond this level even after sending multiple e-mails. To indirectly overcome this limitation, we evaluated whether survey respondents had different demographic characteristics from all the KRS members. In particular, the age distribution, which has the potential to affect the ability to access the email distributed surveys and cause selection bias, was compared between the survey respondents and all KRS members, and there was no statistically significant difference between them (*p* = 0.447, chi-square test, data not shown). Third, our study design was based on expert option rather than prospective clinical trials, which could have produced more concrete results. Summarizing the opinions of retinal specialists in this study cannot provide an opinion that completely ignores any possible existing misconceptions. However, considering the ethical issues and difficulties of conducting clinical trials in patients with retinal diseases in the real world, the results of this study can help cataract surgeons make decisions about diffractive MF-IOL implantation in eyes with underlying retinal diseases until more concrete evidence is established.

Despite these limitations, one merit of this study is that it is the first attempt to collect the opinions of qualified retinal specialists on the conditions for MF-IOL implantation. Retinal specialists are expected to have a realistic view of MF-IOL implantation in eyes with retinal diseases because they can receive continuous feedback through clinical experiences. A second merit is that our suggestions do not merely show dichotomous results between approval and disapproval of MF-IOL implantation; instead, they show varying degrees of approval depending on disease/status and severity. In real-world clinical situations, it is expected that the degree of patient demand for presbyopia correction and the degree of approval of the retinal specialist should be in harmony when an appropriate IOL is being selected for each patient. The results of this study are expected to help cataract surgeons make decisions on appropriate IOL selection in patients with underlying retinal diseases who prefer to correct presbyopia with cataracts.

In conclusion, most of the respondents disapproved of MF-IOL implantation in eyes with retinal diseases involving the macula although varying degrees of disapproval were reported. The results of this study, consisting of the degree of approval for each of several retinal disease categories, are expected facilitate the decision-making process for MF-IOL implantation. The factors that affected the specialists’ opinions were the type of practice and the frequency with which they implanted MF-IOLs.

## Figures and Tables

**Table 1:** Demographics.

Factors	Number (%)
Career duration, *n* (%)	
<10 years in practice	53 (47.7)
≥10 years in practice	58 (52.3)
Type of practice, *n* (%)	
Tertiary hospital	48 (43.2)
General hospital	4 (3.6)
Eye hospital	26 (23.4)
Private clinic	33 (29.7)
Frequency of MF-IOL implantation	
None	38 (34.2)
≤5% of cases	45 (40.5)
>5% of cases	28 (25.2)
Age	
30~39 years old	42 (37.8)
40~49 years old	45 (40.5)
50~59 years old	18 (16.2)
60~69 years old	6 (5.4)
Total	111

MF-IOL, multifocal intraocular lens.

**Table 2:** The degree of approval of the Korean retinal specialists regarding implantation of diffractive multifocal intraocular lenses in eyes with each of retinal disease/status.

Retinal Disease/Status	Descriptive Statistics of Likert Scores		% of Responses	
	Median	Average	Disapproving (1–3)	Permissive (5–7)
Wet AMD	1	1.4	96.4	0.9
PDR, ME (+)	1	1.5	94.6	0.9
NPDR, ME (+)	2	2.0	89.2	4.5
Dry AMD with GA	2	2.1	87.4	5.4
PDR, ME (−)	2	2.4	75.7	10.8
Previous macula-off RD	3	3.0	64.9	19.8
Previous RVO	3	3.2	53.2	21.6
Epiretinal membrane	3	3.4	52.3	30.6
Dry AMD without GA	4	3.6	47.7	35.1
Resolved CSC	4	3.9	42.3	40.5
NPDR, ME (−)	4	4.0	36.9	42.3
High myopia	4	4.3	33.3	49.5
Previous macula-on RD	5	4.4	29.7	53.2
Previous history of barrier laser due to retinal breaks	6	5.8	7.2	82.0
Lattice degeneration	6	5.8	5.4	82.9

AMD, age-related macular degeneration; CSC, central serous chorioretinopathy; GA, geographic atrophy; NPDR, nonproliferative diabetic retinopathy; ME, macular edema; PDR, proliferative diabetic retinopathy; RD, retinal detachment; RVO, retinal vein occlusion.

**Table 3:** Factors that may influence the opinions of retinal specialists.

	Criteria	Total (*n* = 111)	Average Sum of Questionnaire Scores (Mean ± Standard Deviation)	*p*-Value *
Age	30~39 years old	42	53.8 ± 11.7	0.186
	40~49 years old	45	48.2 ± 14.8	
	50~69 years old	24	50.6 ± 16.2	
Career duration	<10 years	53	52.5 ± 12.0	0.236
	≥10 years	58	49.4 ± 15.8	
Type of practice	Tertiary hospital	48	48.0 ± 15.2	0.013 ^†^
	Eye hospital	26	57.9 ± 11.0	
	Private clinic	33	49.8 ± 13.5	
Frequency of MF-IOL implantation	None	38	46.3 ± 12.3	0.021 ^†^
	≤5% of cases	45	51.5 ± 16.6	
	>5% of cases	28	55.9 ± 10.0	

* Mann–Whitney U test or one-way ANOVA; ^†^ Statistically significant (*p* < 0.05); MF-IOL, multifocal intraocular lens.

## Data Availability

Not applicable.

## References

[1] Berdahl J., Bala C., Dhariwal M., Lemp-Hull J., Thakker D., Jawla S. (2020). Patient and Economic Burden of Presbyopia: A Systematic Literature Review. Clin. Ophthalmol..

[2] Ahmad B.U., Shah G.K., Hardten D.R. (2014). Presbyopia-correcting intraocular lenses and corneal refractive procedures: A review for retinal surgeons. Retina.

[3] Klein B.R., Brown E.N., Casden R.S. (2016). Preoperative macular spectral-domain optical coherence tomography in patients considering advanced-technology intraocular lenses for cataract surgery. J. Cataract Refract. Surg..

[4] Chung J.K., Lee H.K., Kim M.K., Kim H.K., Kim S.W., Kim E.C., Kim H.S. (2019). Cataract Surgery Practices in the Republic of Korea: A Survey of the Korean Society of Cataract and Refractive Surgery 2018. Korean J. Ophthalmol..

[5] Health Insurance Review & Assessment Service. https://www.hira.or.kr/bbsDummy.do?pgmid=HIRAA020004000000&brdScnBltNo=4&brdBltNo=11292&pageIndex=1.

[6] de Vries N.E., Nuijts R.M. (2013). Multifocal intraocular lenses in cataract surgery: Literature review of benefits and side effects. J. Cataract Refract. Surg..

[7] Grzybowski A., Kanclerz P., Tuuminen R. (2020). Multifocal intraocular lenses and retinal diseases. Graefes Arch. Clin. Exp. Ophthalmol..

[8] Chen T., Yu F., Lin H., Zhao Y., Chang P., Lin L., Chen Q., Zheng Q., Zhao Y.E., Lu F. (2016). Objective and subjective visual quality after implantation of all optic zone diffractive multifocal intraocular lenses: A prospective, case-control observational study. Br. J. Ophthalmol..

[9] Pham T.Q., Wang J.J., Rochtchina E., Maloof A., Mitchell P. (2004). Systemic and ocular comorbidity of cataract surgical patients in a western Sydney public hospital. Clin. Exp. Ophthalmol..

[10] Riley A.F., Malik T.Y., Grupcheva C.N., Fisk M.J., Craig J.P., McGhee C.N. (2002). The Auckland cataract study: Co-morbidity, surgical techniques, and clinical outcomes in a public hospital service. Br. J. Ophthalmol..

[11] Montes-Mico R., Espana E., Bueno I., Charman W.N., Menezo J.L. (2004). Visual performance with multifocal intraocular lenses: Mesopic contrast sensitivity under distance and near conditions. Ophthalmology.

[12] Tanabe H., Tabuchi H., Shojo T., Yamauchi T., Takase K. (2020). Comparison of visual performance between monofocal and multifocal intraocular lenses of the same material and basic design. Sci. Rep..

[13] Cugati S., Mitchell P., Rochtchina E., Tan A.G., Smith W., Wang J.J. (2006). Cataract surgery and the 10-year incidence of age-related maculopathy: The Blue Mountains Eye Study. Ophthalmology.

[14] Braga-Mele R., Chang D., Dewey S., Foster G., Henderson B.A., Hill W., Hoffman R., Little B., Mamalis N., Oetting T. (2014). Multifocal intraocular lenses: Relative indications and contraindications for implantation. J. Cataract Refract. Surg..

[15] Klein R., Klein B.E. (2013). The prevalence of age-related eye diseases and visual impairment in aging: Current estimates. Investig. Ophthalmol. Vis. Sci..

[16] Alio J.L., Plaza-Puche A.B., Fernandez-Buenaga R., Pikkel J., Maldonado M. (2017). Multifocal intraocular lenses: An overview. Surv. Ophthalmol..

[17] Kawamura R., Inoue M., Shinoda K., Bissen-Miyajima H. (2008). Intraoperative findings during vitreous surgery after implantation of diffractive multifocal intraocular lens. J. Cataract Refract. Surg..

[18] Inoue M., Bissen-Miyajima H., Yoshino M., Suzuki T. (2009). Wavy horizontal artifacts on optical coherence tomography line-scanning images caused by diffractive multifocal intraocular lenses. J. Cataract Refract. Surg..

[19] Yoshino M., Inoue M., Kitamura N., Bissen-Miyajima H. (2010). Diffractive multifocal intraocular lens interferes with intraoperative view. Clin. Ophthalmol..

[20] Dias-Santos A., Costa L., Lemos V., Anjos R., Vicente A., Ferreira J., Cunha J.P. (2015). The impact of multifocal intraocular lens in retinal imaging with optical coherence tomography. Int. Ophthalmol..

[21] Hadayer A., Jusufbegovic D., Schaal S. (2017). Retinal detachment repair through multifocal intraocular lens- overcoming visualization challenge of the peripheral retina. Int. J. Ophthalmol..

[22] Watanabe T., Watanabe A., Nakano T. (2020). Suitability of Different Observational Lenses for Viewing the Macular Area Through Multifocal Intraocular Lenses in a Model of the Human Eye. Clin. Ophthalmol..

[23] Lee J.Y., Joo K., Park S.J., Woo S.J., Park K.H. (2021). Epiretinal membrane surgery in patients with multifocal versus monofocal intraocular lenses. Retina.

[24] Rementeria-Capelo L.A., Garcia-Perez J.L., Contreras I., Blazquez V., Ruiz-Alcocer J. (2021). Impact of Trifocal and Trifocal Toric Intraocular Lenses on Spectral-domain OCT Retinal Measurements. J. Glaucoma.

[25] Farid M., Chak G., Garg S., Steinert R.F. (2014). Reduction in mean deviation values in automated perimetry in eyes with multifocal compared to monofocal intraocular lens implants. Am. J. Ophthalmol..

[26] Logothetis H.D., Feder R.S. (2019). Which intraocular lens would ophthalmologists choose for themselves?. Eye.

